# Evaluation of corneal intrastromal voriconazole injection as an adjuvant approach in recalcitrant fungal keratitis

**DOI:** 10.1186/s12348-026-00599-6

**Published:** 2026-06-23

**Authors:** Khaled El Ghonemy Said, Nada Mohammed Aggour, Ahmed Ibrahim Basiony

**Affiliations:** 1 Department of Ophthalmology, Faculty of Medicine, Menoufia University, Shebin El-Kom, Menoufia Egypt; 2 Ophthalmology Eye Hospital, Shebin El Kom, Menoufia Egypt

**Keywords:** Fungal keratitis, Intrastromal voriconazole, AS-OCT, Recalcitrant, Ulcer

## Abstract

**Background:**

Fungal keratitis is a sight-threatening condition that accounts for approximately 30–40% of keratitis cases in developing countries. Its management remains challenging despite the availability of various antifungal agents, highlighting the potential role of targeted drug delivery in recalcitrant cases.

**Purpose:**

To evaluate the efficacy of corneal intrastromal voriconazole (ISV) injection in the management of fungal keratitis unresponsive to conventional antifungal therapy.

**Methods:**

This prospective interventional case series included 21 eyes of 21 patients with smear-positive fungal keratitis that failed to respond to at least two weeks of topical antifungal therapy. All patients underwent detailed ophthalmological examination and ulcer assessment using anterior segment optical coherence tomography (AS-OCT). Intrastromal voriconazole (50 µg/0.1 mL) was administered circumferentially around the ulcer. Treatment response was evaluated through serial follow-up, including assessment of ulcer size, infiltrate extent, hypopyon level, presence of satellite lesions, and best-corrected visual acuity (BCVA).

**Results:**

The mean age of the patients was 57.19 ± 6.74 years, with a predominance of males (76.2%). Most patients were from rural areas (76.2%) and had a history of vegetative trauma (71.4%). The mean ulcer size was 4.64 ± 0.95 mm, and the mean infiltrate size was 6.43 ± 1.29 mm. Hypopyon was present in 57.1% of cases, and the mean ulcer depth measured by AS-OCT was 257.14 ± 68.12 μm. Aspergillus was the most isolated organism (52.4%). BCVA improved significantly from 2.71 ± 0.46 LogMAR at baseline to 1.34 ± 0.68 at 3 months (*P* < 0.001). Complete resolution was achieved in 19 patients (90.5%). Fourteen patients (66.7%) responded to a single injection, while five (23.8%) and two (9.5%) required two and three injections, respectively. Most cases (84.2%) resolved within 2–4 weeks. Two patients (9.5%) showed disease progression and required therapeutic penetrating keratoplasty and were excluded from the final analysis.

**Conclusion:**

Intrastromal voriconazole injection appears to be an effective adjunctive treatment for recalcitrant fungal keratitis, improving clinical outcomes and potentially reducing the need for therapeutic or tectonic keratoplasty.

## Introduction

Fungal keratitis is a significant cause of visual impairment, particularly in developing countries, where delayed presentation and limited access to appropriate treatment often result in poor outcomes [1]. Its higher prevalence in tropical and subtropical regions is largely related to agricultural activities, as well as hot and humid environmental conditions. In addition, limited public awareness and restricted healthcare resources further contribute to the disease burden in these settings [2]. Several risk factors have been associated with fungal keratitis, including ocular trauma with vegetative matter, the use of contaminated contact lenses, pre-existing ocular surface disorders, immunosuppression, and inappropriate use of topical corticosteroids or antibiotics [3].

Despite recent therapeutic advances, management remains difficult due to the poor corneal penetration, limited coverage of topical antifungal agents, and the reported ocular surface toxicity. Consequently, the condition may progress to severe complications such as descemetocele formation, corneal melting, perforation, endophthalmitis, and ultimately vision loss [4]. Keratoplasty may ultimately be required in cases of deep stromal fungal keratitis that fail to respond to medical therapy. However, its success is limited by factors such as graft rejection, recurrence of infection, and the restricted availability of donor corneas, particularly in developing countries [4].

Natamycin is widely regarded as the first-line treatment for filamentous fungal keratitis due to its broad-spectrum activity, favorable safety profile, and chemical stability. However, its efficacy is limited by poor penetration through an intact corneal epithelium, often necessitating epithelial debridement to enhance drug delivery [5, 6]. Recently, newer antifungal agents such as voriconazole, caspofungin, and posaconazole have demonstrated improved safety profiles and enhanced corneal penetration, with outcomes that may surpass those of conventional therapies. Moreover, targeted drug delivery approaches, particularly intrastromal injection, have shown promising results by achieving higher local drug concentrations directly at the site of infection [7].

The present study was conducted to evaluate the clinical and structural outcomes of intrastromal voriconazole injection in recalcitrant fungal keratitis. In addition to assessing efficacy, the study aimed to characterize treatment response patterns and the need for repeated injections, with correlation to structural changes documented by anterior-segment optical coherence tomography (AS-OCT).

## Methods

This prospective interventional case series was conducted at the Cornea Unit at the Department of Ophthalmology, Menoufia University Hospital, and a private eye center between February 2024 and July 2025. The study included 21 patients with recalcitrant fungal keratitis. The study was approved by the institutional ethics committee (IRB 2/2024 OPHT12) and adhered to the tenets of the Declaration of Helsinki. All participants provided informed consent after a thorough explanation of the procedure.

Eligible patients were older than 19 years, had smear- and culture-confirmed fungal keratitis, and failed to respond to conventional topical antifungal therapy for more than two weeks. Patients with mixed infections, scleral involvement, impending perforation, signs of endophthalmitis, or known hypersensitivity to the study drug were excluded. Additional exclusion criteria included patients younger than 18 years, those with a single functional eye, or visual acuity less than 6/60 in the fellow eye.

At presentation, all patients underwent comprehensive ophthalmological evaluation, including detailed history taking, best-corrected visual acuity (BCVA) assessment, and slit-lamp biomicroscopy. Clinical parameters recorded included ulcer size, extent of stromal infiltration, presence of satellite lesions, and hypopyon level. Ulcer size was determined based on the maximum diameter. Structural assessment was further performed using anterior segment optical coherence tomography (AS-OCT) (Carl Zeiss Cirrus 5000, Meditec Inc., Germany). Corneal scraping was performed under topical anesthesia (0.5% proparacaine hydrochloride) and subjected to microbiological evaluation, including potassium hydroxide (KOH) staining, Gram staining, and culture on blood agar, chocolate agar, and Sabouraud dextrose agar.

Following confirmation of diagnosis, all patients received topical antifungal therapy consisting of 5% natamycin sulfate and 1% voriconazole eye drops administered hourly for two weeks. Clinical response was evaluated by slit-lamp examination. Lack of change in ulcer or infiltrate size was classified as “no improvement,” while an increase of ≥ 20% in size or depth was considered “progression.” Healing was defined as a reduction of more than 20% in ulcer and infiltrate size compared to baseline.

**Fig. 1:**
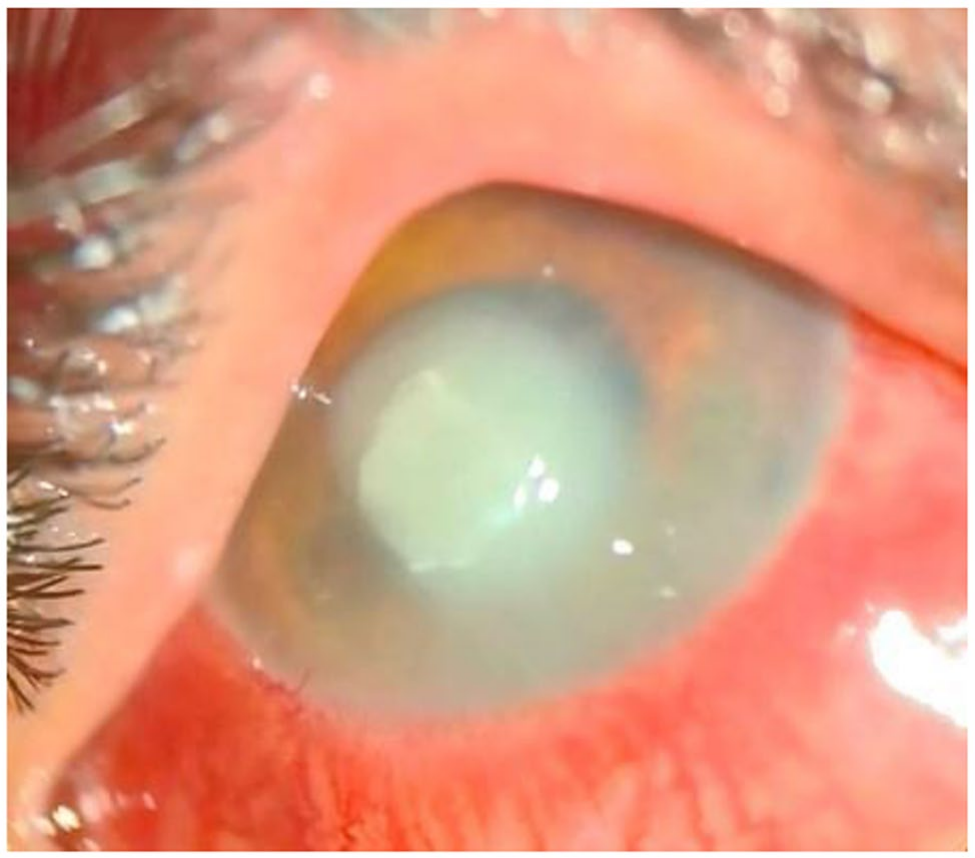
A case of recalcitrant Fungal Keratitis immediately after ISV injection

Patients who showed no improvement or progression after two weeks received intrastromal voriconazole injection (50 µg/0.1 mL), administered circumferentially around the fungal infiltrate. Voriconazole was prepared by reconstituting 200 mg of lyophilized powder with 19 mL of lactated Ringer’s solution to obtain a concentration of 10 mg/mL, followed by further dilution to achieve a final concentration of 0.5 mg/mL (50 µg/0.1 mL). The solution was loaded into a 1 mL tuberculin syringe with a 30-gauge needle. Under peribulbar anesthesia and strict aseptic conditions, the needle was inserted obliquely through the clear cornea to reach the mid-stromal level surrounding the infiltrate. The drug was injected in five divided doses around the lesion to create a circumferential barrier of medication. (Fig. 1) Any intraoperative complications were recorded.

Following injection, patients continued their topical antifungal regimen and were examined daily for the first three days, then at one week, and subsequently every two weeks for up to three months or until complete healing. Clinical response was assessed by changes in BCVA, ulcer and infiltrate size, hypopyon level, and presence of satellite lesions. Resolution was defined as complete epithelial healing with disappearance of infiltrates and formation of a corneal scar. Topical antifungal therapy was continued for at least one week after complete resolution.

Repeated intrastromal injection was performed in cases showing no improvement within 3–7 days. Treatment failure was defined as progression of infiltration by more than 20% despite three injections or the occurrence of corneal perforation, in which case patients were scheduled for amniotic membrane grafting or tectonic/therapeutic penetrating keratoplasty.

### Statistical analysis of the data

Data were analyzed using IBM SPSS Statistics version 20.0 (IBM Corp., Armonk, NY, USA). Qualitative variables were presented as frequencies and percentages. Normality of distribution was assessed using the Shapiro–Wilk test. Quantitative data were expressed as range (minimum–maximum), mean ± standard deviation, median, and interquartile range (IQR). Statistical significance was set at a p-value ≤ 0.05.

## Results

A total of 21 patients with recalcitrant fungal keratitis were included in this study. The cohort comprised 16 males (76.2%) and 5 females (23.8%), with a mean age of 57.19 ± 6.74 years (range: 43–67 years). Most patients were from rural areas (76.2%), and a history of vegetative ocular trauma was reported in 71.4% of cases. On slit-lamp examination, all patients demonstrated stromal involvement ranging from the anterior one-third to the anterior two-thirds of the cornea and presented with pain, redness, and photophobia.

The mean corneal ulcer size was 4.64 ± 0.95 mm (range: 3.0–6.0 mm), while the mean infiltrate size was 6.43 ± 1.29 mm (range: 4.5–8.5 mm). Hypopyon was present in 57.1% of patients. AS-OCT assessment revealed a mean depth of stromal infiltration of 257.14 ± 68.12 μm (range: 160–365 μm). Microbiological analysis confirmed fungal infection in all cases, with *Aspergillus* being the most commonly isolated organism (52.4%), followed by *Fusarium* and *Candida* (23.8% each), as shown in Table 1.

**Table 1:** Microbiological profile of fungal keratitis cases

	No.	%
Fungus KOH (hyphea)	21	100.0
Culture		
Aspergillus	11	52.4
Candida	5	23.8
Fusarium	5	23.8

**Fig. 2:**
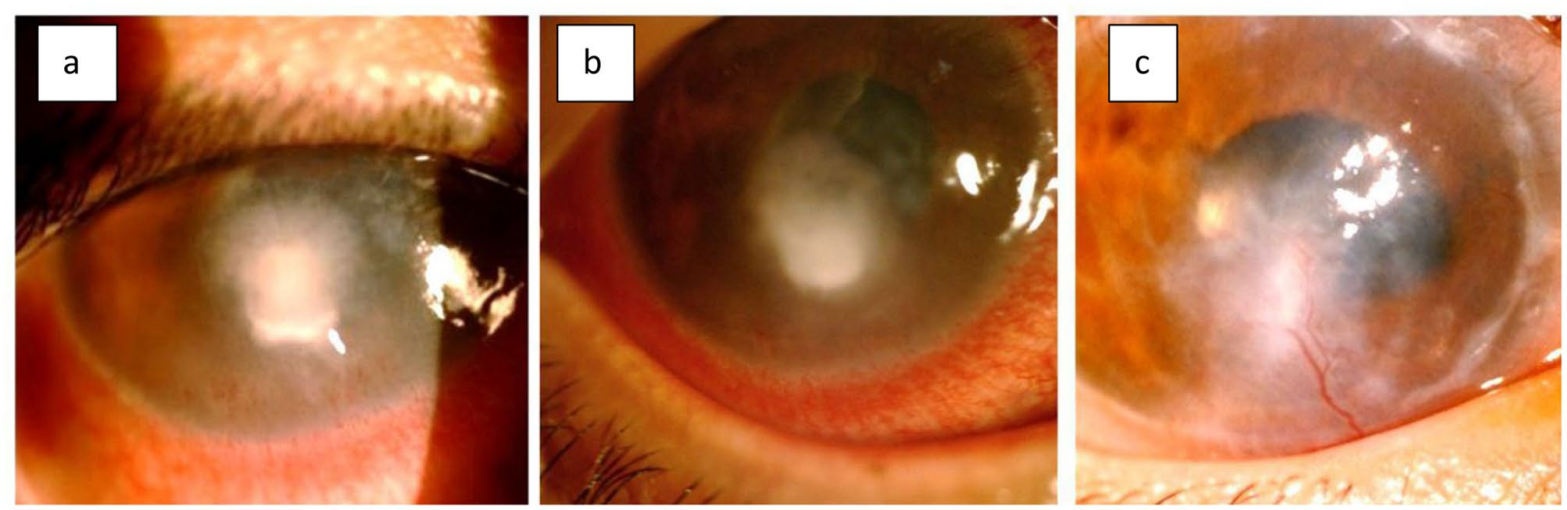
Clinical course of a case with paracentral fungal keratitis (approximately 3.5 mm in maximum diameter) following intrastromal voriconazole (ISV) injection: (**a**) day 1 post-injection, (**b**) 1 week, and (**c**) 1 month, showing progressive healing

**Fig. 3:**
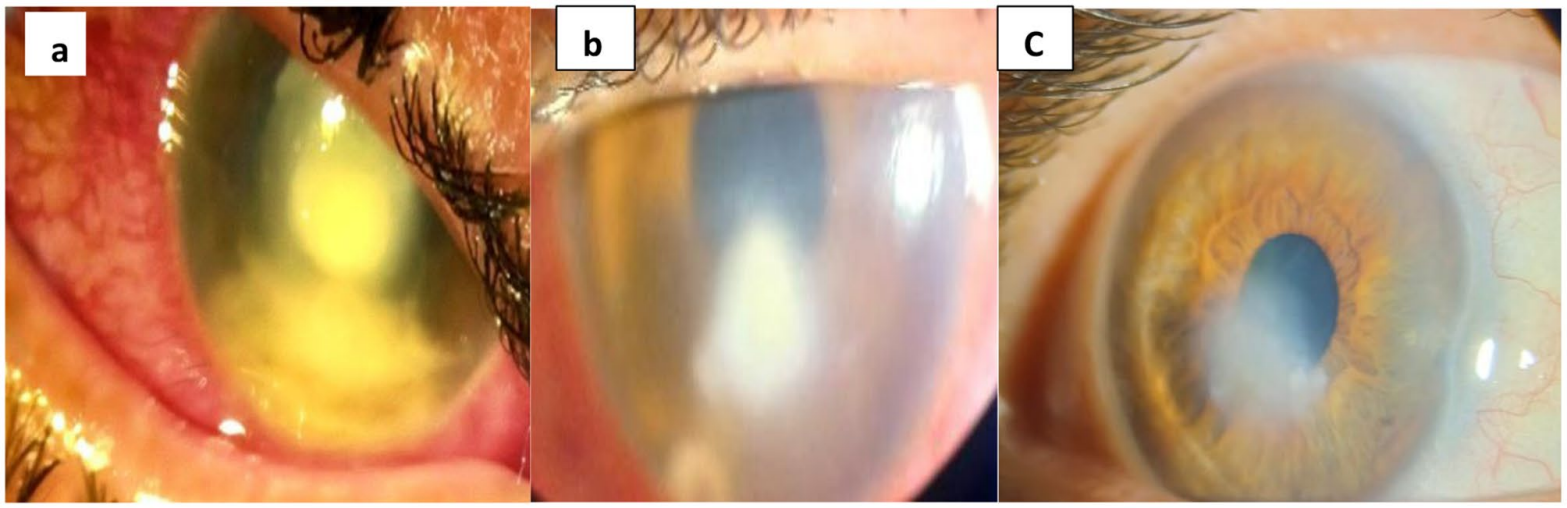
Clinical course of a case with central corneal fungal ulcer (approximately 4 mm in maximum diameter) associated with a 3 mm hypopyon following intrastromal voriconazole (ISV) injection: (**a**) day 1 post-injection, (**b**) 1 month, and (**c**) 3 months, demonstrating progressive resolution

**Fig. 4:**
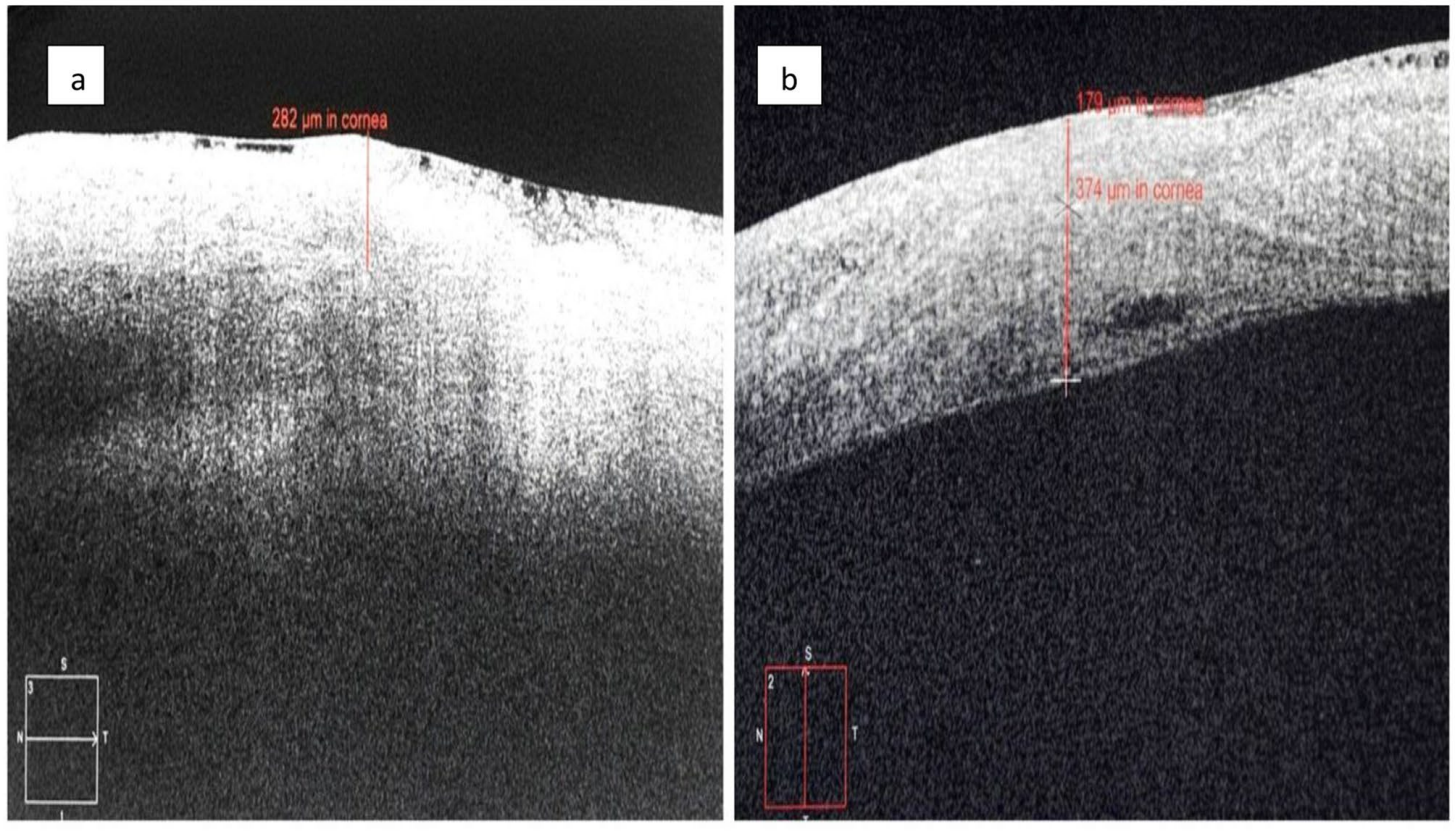
Anterior segment optical coherence tomography (AS-OCT) images demonstrating stromal healing in fungal keratitis following intrastromal voriconazole (ISV) injection: (**a**) pre-treatment showing infiltration depth of 282 μm, and (**b**) 1 month post-injection showing reduction to 179 μm

Following intrastromal voriconazole (ISV) injection, complete resolution was achieved in 19 patients (90.5%), while two patients (9.5%) showed disease progression leading to corneal perforation and required therapeutic penetrating keratoplasty. Fourteen patients (66.7%) responded to a single injection, whereas five (23.8%) and two (9.5%) required two and three injections, respectively (Table 2). Healing was characterized by resolution of symptoms, epithelial closure, reduction in hypopyon, regression of stromal infiltration, and subsequent scar formation, which was clinically observed and illustrated in (Figs. 2 and 3), and further confirmed by AS-OCT findings (Fig. 4).

**Table 2:** Clinical outcomes and injection frequency of intrastromal voriconazole

Healing Time	Number of Patients	Percentage (%)
1 week	1	4.8%
2–4 weeks	16	76.2%
> 4 weeks	2	9.5%
Not healed (scheduled for PK)	2	9.5%
**No. of injections**	**No.**	**%**
1	14	66.7
2	5	23.8
3	2	9.5

Among the successfully treated cases, complete resolution occurred within 1 week in 1 patient (5.3%), within 2–4 weeks in the majority (84.2%), and after more than 4 weeks in 2 patients (10.5%) who required three injections at one-week intervals (Table 2). Visual acuity improved progressively over time; mean BCVA improved from 2.71 ± 0.46 LogMAR at presentation to 2.43 ± 0.56 LogMAR at 1 month (*p* = 0.28) and to 1.34 ± 0.68 LogMAR at 3 months (*p* < 0.001). Overall, 90.4% of patients demonstrated visual improvement, with 42.9% achieving 6/60 or better at 3 months, as detailed in Table 3. The two patients who required keratoplasty had poor visual outcomes, indicating that anatomical resolution was generally associated with meaningful functional improvement.

**Table 3:** Visual outcomes following intrastromal voriconazole

BCVA	Presentation	1 Month	3 Months
HM	15 (71.4%)	10 (47.6%)	2 (9.5%)
CF to < 6/60	6 (28.6%)	11 (52.4%)	10 (47.6%)
≥ 6/60	0	0	9 (42.9%)
Mean BCVA (LogMAR ± SD)	2.71 ± 0.46	2.43 ± 0.56	1.34 ± 0.68
Median (IQR)	3.0 (2.0–3.0)	2.0 (2.0–3.0)	1.10 (1.0–1.70)
p-value vs. baseline	—	0.280	< 0.001

Abbreviation: HM: Hand Motion CF: Counting Fingers, IQR: Inter quartile range, SD: Standard deviation

## Discussion

Fungal keratitis remains a major cause of ocular morbidity, particularly in developing regions where environmental and socioeconomic factors contribute to its high prevalence. A recent study from the Egyptian Delta reported that fungal keratitis accounts for up to two-thirds of microbial keratitis cases, with a proportion involving mixed infections [2]. The management of recalcitrant fungal keratitis continues to be challenging due to the limited stromal penetration, surface toxicity, and restricted spectrum of currently available topical antifungal agents [8]. In this context, targeted drug delivery through intrastromal injection has been proposed as a strategy to achieve higher local drug concentrations and improve treatment outcomes [9].

In the present study, ISV was used as an adjunctive therapy in cases unresponsive to conventional treatment, yielding a high success rate of 90.5%. Most cases demonstrated clinical resolution within 2–4 weeks, with a smaller proportion requiring repeated injections. These findings are consistent with previous reports, such as Bhirud et al., who documented favorable outcomes using ISV in resistant fungal keratitis [4]. Similarly, several studies have supported the safety and efficacy of intrastromal voriconazole in recalcitrant cases [7, 9–12]. Mahmoud et al. also reported superior outcomes with ISV compared to topical therapy alone, further supporting its role as an effective adjunct [13]. Moreover, Saluja et al. demonstrated higher success rates with intrastromal voriconazole compared to other antifungal agents, reinforcing its therapeutic advantage in resistant infections [14].

Microbiological findings in this study revealed that Aspergillus species were the most commonly isolated organisms, followed by Fusarium and Candida. This distribution aligns with several previous studies reporting Aspergillus as the predominant pathogen in fungal keratitis [2, 9, 13]. However, variability in pathogen prevalence has been noted, with other studies identifying Fusarium as the leading organism in certain populations [10, 15]. This variation may partly explain differences in treatment outcomes, as response to voriconazole has been shown to depend on fungal species. Indeed, some studies have reported reduced efficacy of ISV in filamentous infections, particularly those caused by Fusarium, which exhibit lower susceptibility to azole antifungals [16, 17]. Conversely, other reports suggest that voriconazole may still provide favorable outcomes in such infections, highlighting the ongoing debate regarding organism-specific effectiveness [15]. These discrepancies emphasize the need for individualized treatment strategies based on microbiological profile.

An important observation in the present study was that one-third of patients required repeated injections to achieve complete resolution. This finding is in agreement with previous reports indicating that multiple injections may be necessary to maintain adequate drug levels within the corneal stroma [9]. The pharmacokinetics of intrastromal voriconazole remain an area of active investigation, with some studies suggesting that combined approaches, such as intrastromal and intracameral administration, may improve outcomes in severe infections [18]. Additionally, combined antifungal strategies have been explored to enhance efficacy and reduce recurrence rates [19]. These findings suggest that while ISV is effective, optimization of dosing protocols and delivery methods is still required.

Visual outcomes in this study showed significant improvement over time, with 90.4% of patients demonstrating visual gain and 42.9% achieving visual acuity of 6/60 or better. These results are comparable to previous studies, such as Sharma et al., who reported visual improvement in the majority of treated eyes [9], and Kalaiselvi et al., who documented similar functional outcomes despite variability related to fungal species [7]. The improvement in visual acuity observed in our study likely reflects both anatomical resolution of infection and prevention of further stromal damage, particularly in cases treated early with intrastromal therapy.

Regarding safety, ISV was well tolerated in our study, with no direct injection-related complications observed. Only two cases progressed to corneal perforation, necessitating therapeutic keratoplasty. Previous studies have reported potential complications such as stromal edema, scarring, and mechanical damage related to injection technique [7, 9, 20], although direct drug toxicity has not been consistently demonstrated [16]. These findings underscore the importance of proper case selection, careful injection technique, and close follow-up when using this modality.

Despite the promising results, this study has several limitations. The relatively small sample size may limit the generalizability of the findings, and the absence of a control or comparative group restricts the ability to directly evaluate the superiority of intrastromal voriconazole over other treatment modalities. Furthermore, optimal dosing strategies and injection frequency remain to be established. Future studies with larger cohorts and randomized designs are needed to better define the role of ISV and to standardize treatment protocols.

## Conclusion

ISV is an effective and practical adjunctive modality in the management of recalcitrant fungal corneal ulcers. Given the limited availability and suboptimal stromal penetration of topical antifungal agents, intrastromal delivery of voriconazole offers targeted therapy that enhances drug bioavailability at the site of infection. This approach may accelerate ulcer healing, reduce the risk of complications, and decrease the need for surgical interventions such as therapeutic keratoplasty.

## Data Availability

The data sets used and/or analyzed during the current study are available from the corresponding author on reasonable request.
